# Snowballs and Icicles in Susac’s Syndrome

**DOI:** 10.5334/jbsr.2441

**Published:** 2021-04-23

**Authors:** Jesper Dierickx, Filip Vanhoenacker, Virginie Merckaert

**Affiliations:** 1 AZ Sint-Maarten, BE; 2 Faculty of Medicine, University of Ghent, BE; 3 AZ Sint-Maarten and University (Hospital) Antwerp/Ghent, BE

**Keywords:** Magnetic Resonance Imaging, Susac Syndrome

## Abstract

**Teaching Point:** Snowball-like and icicle-like lesions in the corpus callosum suggest Susac Syndrome.

## Case Presentation

A 45-year-old man presents with headache and photophobia over several weeks and word-finding difficulties, multifocal paresthesia, vertigo, and right-sided hearing loss for several days. He has no relevant past medical history.

**Figure 1:**
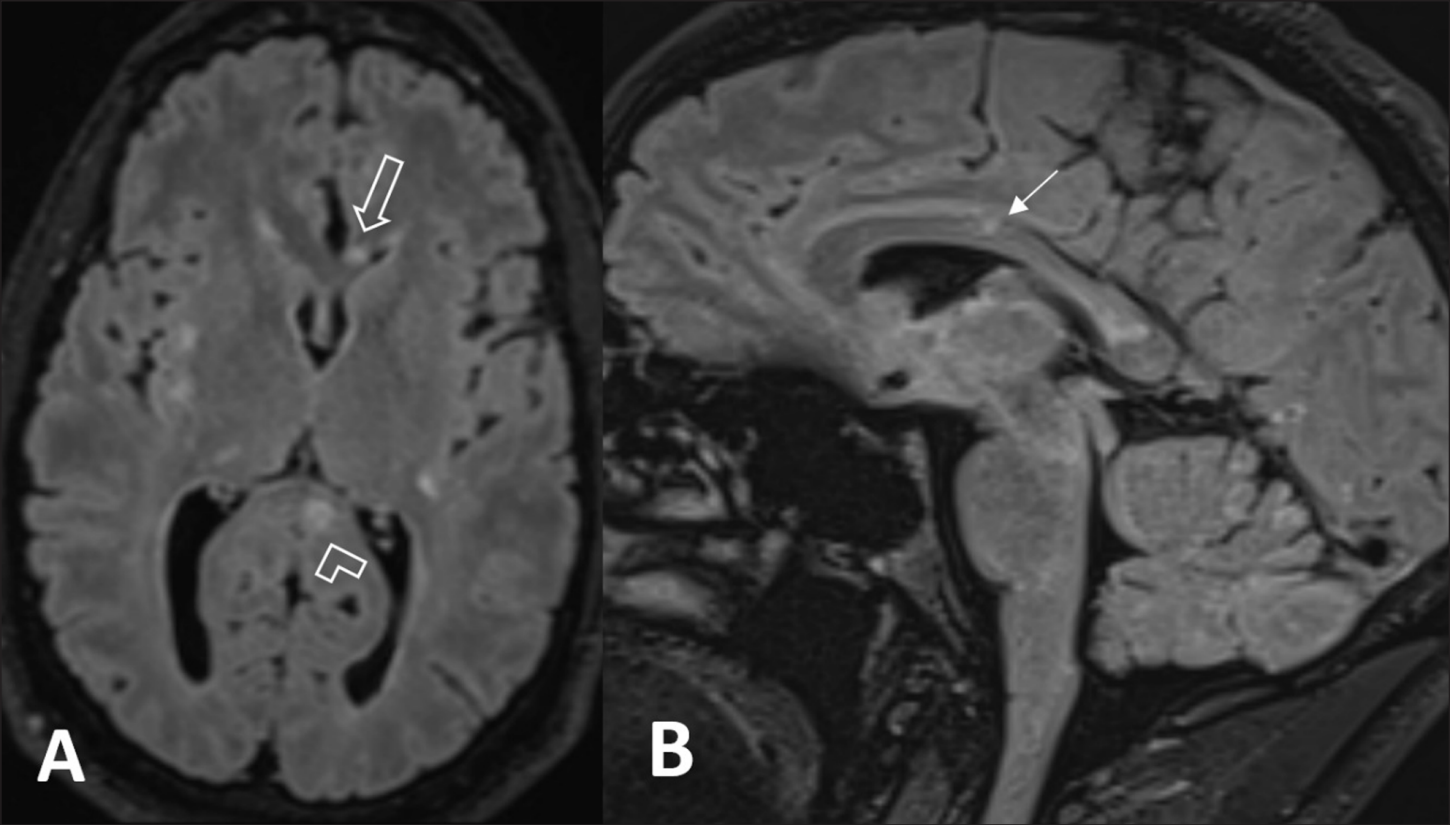


**Figure 2:**
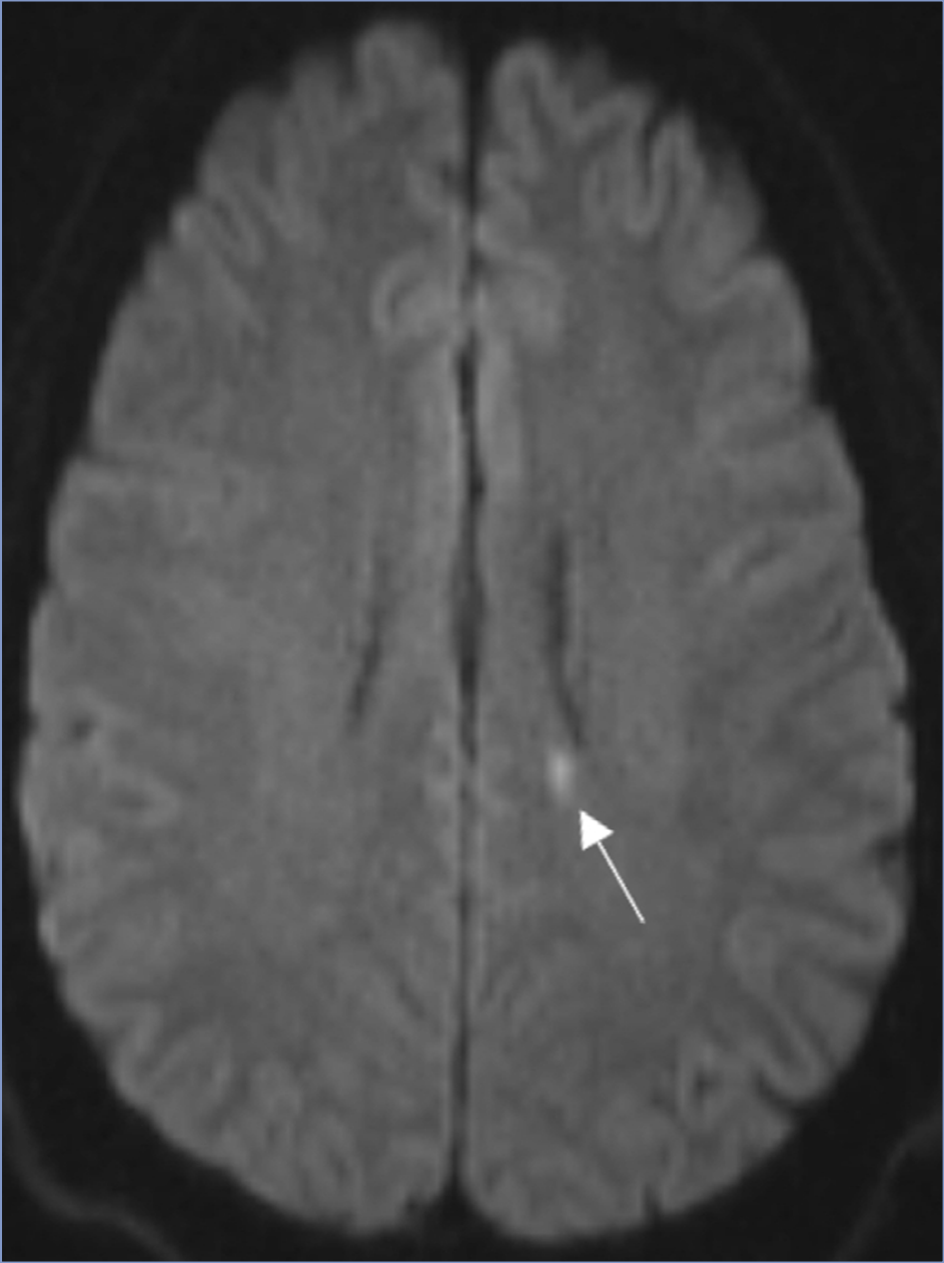


**Figure 3:**
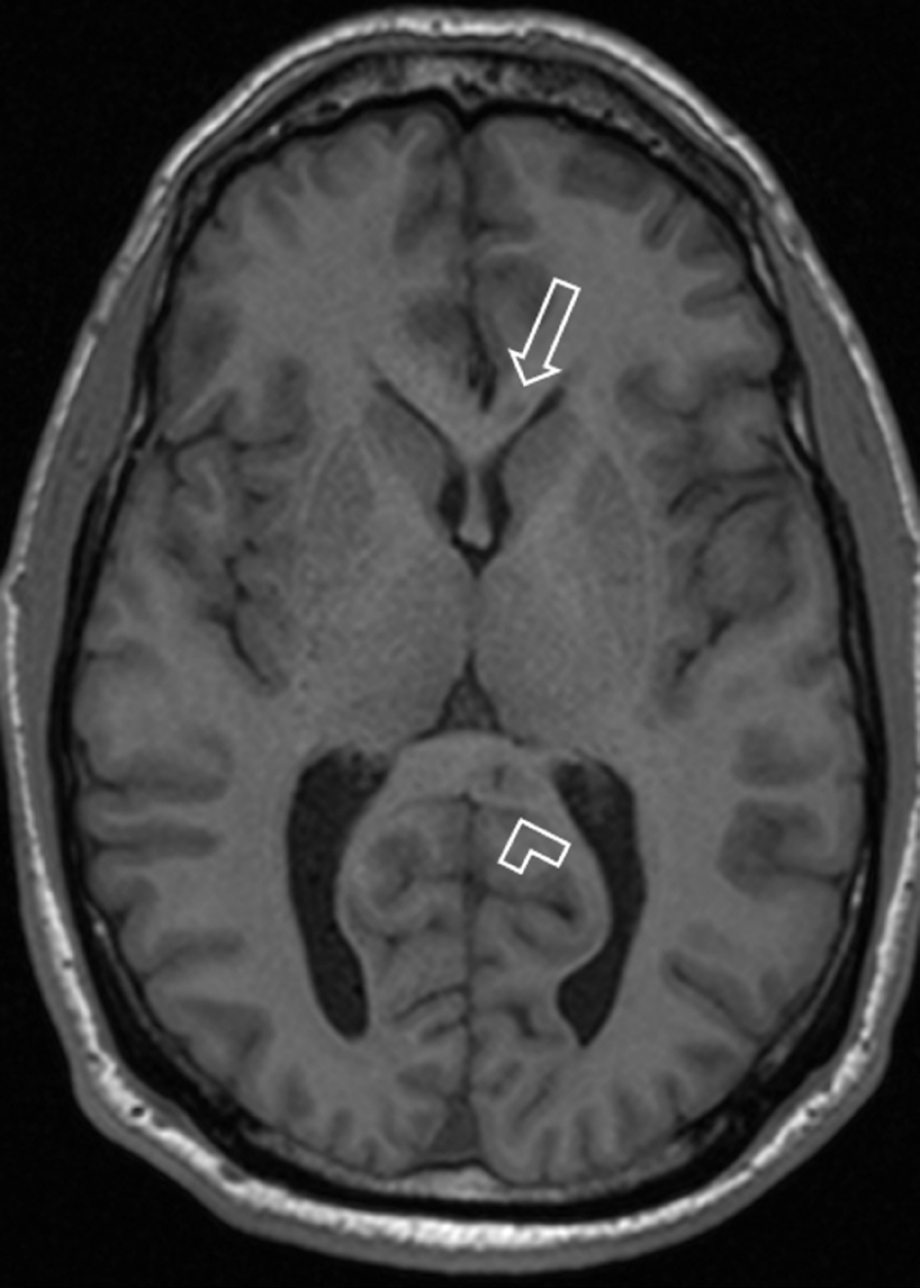


Magnetic resonance imaging (MRI) shows multiple hyperintense white matter lesions on fluid-attenuated inversion-recovery (FLAIR) sequence (***Figure 1***). Round lesions of the corpus callosum (CC) are located in the genu and the splenium (***Figure 1a***; void arrow and arrowhead). Another wedged-shaped lesion extends from the roof in the truncus, without contacting the CC base (***Figure 1b***; white arrow). Diffusion-weighted imaging (D-WI) shows a diffusion restrictive lesion in the truncus (***Figure 2***; white arrow). The genu and splenium lesions are hypo-intense on T1-WI (***Figure 3***; void arrow and arrowhead).

Retinal fluorescein angiography (FA) shows peripheral retinal arterial occlusion with arterial plaques. Audiogram confirms right-sided sensorineural hearing loss.

Based on the combination of clinical, FA, audiogram findings and imaging features, the diagnosis of Susac syndrome was made.

## Comment

Susac syndrome (SS) is a rare disease, mainly affecting women between 20 and 40 years old. It is typically characterized by a clinical triad of encephalopathy, branch retinal artery occlusion, and sensorineural hearing loss. In many cases, this triad is not completely present at diagnosis and occurs metachronously. The diagnosis is based on the combination of clinical findings, FA findings, and MRI features. SS is presumably caused by auto-immune mediated micro-infarctions in the brain, retina, and inner ear. The clinical course is mostly self-limiting and monophasic. In some patients, the disease is highly unpredictable and may have a polycyclic, relapsing, or chronic continuous course [1].

MRI is the preferred imaging technique for diagnosis of brain involvement. T2-WI show small, multifocal hyperintense lesions of 3–7 mm. The CC is invariably involved. These CC lesions have a characteristic ‘snowball-like’ or ‘icicle-like’ morphology. ‘Snowball-like’ lesions have a round morphology and a central location in the CC. ‘Icicle-like’ lesions are wedge-shaped and extend from the roof of the CC without contacting the base of the CC. Other, periventricular located lesions are non-specific for SS. Diffusion restrictive lesions indicate SS micro-infarcts in the acute phase. Leptomeningeal enhancement may be present, especially in fulminant cases. CC atrophy is possible. MS and ADEM are the most important differential diagnoses. In contrast to SS, MS and ADEM lesions in the CC are typically ovoid and predominately located at the base and septal interface. Leptomeningeal enhancement and deep gray matter involvement are uncommon in MS and ADEM [1].

An early, aggressive immunosuppressive treatment is indicated to prevent damage. However, an optimal immunosuppressant regimen has not been defined [1].
